# Informal and formal mental health: preliminary qualitative findings

**DOI:** 10.3402/ijch.v72i0.21203

**Published:** 2013-08-05

**Authors:** Linda O'Neill, Serena George, Corinne Koehn, Blythe Shepard

**Affiliations:** 1 Counselling Program, School of Education, University of Northern British Columbia, Prince George, British Columbia, Canada; 2 Counselling Program, University of Lethbridge, Lethbridge, Alberta, Canada

**Keywords:** northern, mental health, formal and informal practitioners, qualitative research

## Abstract

**Background:**

Northern-based research on mental health support, no matter the specific profession, helps to inform instruction of new practitioners and practitioners already working in rural or isolated conditions. Understanding the complexities of northern mental health support not only benefits clients and practitioners living in the North, but also helps prepare psychologists and counsellors preparing to work in other countries with large rural and isolated populations. The qualitative phase is part of a multi-year research study on informal and formal mental health support in northern Canada involving the use of qualitative and quantitative data collection and analysis methods.

**Objective:**

The main objective of the qualitative phase interviews was to document in-depth the situation of formal and informal helpers in providing mental health support in isolated northern communities in northern British Columbia, northern Alberta, Yukon and Northwest Territories (NWT). The intent of in-depth interviews was to collect descriptive information on the unique working conditions of northern helping practitioners for the development of a survey and subsequent community action plans for helping practitioner support.

**Design:**

Twenty participants in northern BC, Yukon and NWT participated in narrative interviews. Consensual qualitative research (CQR) was used in the analysis completed by 7 researchers. The principal researcher and research associate then worked through all 7 analyses, defining common categories and themes, and using selections from each researcher in order to ensure that everyone's analysis was represented in the final consensual summary.

**Results:**

The preliminary results include 7 main categories consisting of various themes. Defining elements of northern practice included the need for generalist knowledge and cultural sensitivity. The task of working with and negotiating membership in community was identified as essential for northern mental health support. The need for revised codes of ethics relevant to the reality of northern work was a major category, as was insight on how to best sustain northern practice.

**Conclusion:**

Many of the practitioners who participated in this study have found ways to overcome the biggest challenges of northern practice, yet the limitations of small populations and lack of resources in small communities to adequately address mental health support were identified as existing. Empowering communities by building community capacity to educate, supervise and support formal and informal mental health workers may be the best approach to overcoming the lack of external resources.

In northern British Columbia, Yukon and Northwest Territories (NWT), communities, access to mental health specialists is severely restricted due to the distance from larger centres. The difficult task of providing mental health services and support in remote northern communities is undertaken by both formal and informal mental health practitioners due to geographical, cultural, community and economic necessity (1–3). Environment determines lifestyle in the North, with geography influencing practice (4), whether that practice is counselling, psychology, social work, nursing or other helping professions. Features of geography and weather add physical isolation to professional and sometimes personal isolation inherent in mental health support in communities with small populations.

Canada's northern region is very sparsely populated with only about 101,310 individuals living in a vast area larger than Western Europe (5). About 69% of the population of the Yukon, NWT and Nunavut is of Aboriginal descent, and these 3 territories each have a greater proportion of Aboriginal inhabitants than any of Canada's provinces, with Yukon having the largest percentage of non-Aboriginal inhabitants (6).

Mental health service provision is challenging throughout the North due to geographical isolation and the resulting remoteness of communities. Transportation difficulties, small community populations and demanding practice conditions make the recruitment of service providers difficult. Mental health services along with other social services have also been cut-back or eliminated in many communities due to fiscal issues experienced by provincial and federal governments, resulting in more responsibility and stress on the few remaining formal helping practitioners and on informal helping support such as lay counsellors, elders, family members and other community helpers. The literature suggests that an understanding of isolated northern cultures is essential for competent practice in such settings (7). This understanding includes the influence of cultural diversity, work and economic factors on the social psychology of northern communities, all topics required for a sound knowledge base (4) for various practitioners, both informal and formal.

## Significance

This study examined the issues related to northern practice experienced by formal and informal helping practitioners in providing mental health support. The existing research on specific professions and northern practice has presented the various challenges of mental health and wellness work in northern communities including geographical and professional isolation issues. However, there is a lack of “proactive literature” (4) on sustaining supports and future vision for northern mental health practice, looking at opportunities rather than challenges (8). The main focus of this study is how practitioners sustain their practice, and what supports are currently available to help them with longevity in their work in the North.

## Research questions

In order to better understand mental health and wellness support in the North, the following questions informed the qualitative phase of the research:

### Principle research question

What are the life-career issues, supports, challenges and barriers for formal and informal helping practitioners in northern communities? For the qualitative phase, this question was simplified and broadened to allow participants to begin the interview where they wanted to start: What is your experience of providing informal or formal mental health and wellness support in northern communities?

### Secondary questions

- What supports and resources would enhance the effectiveness and longevity of such workers? and
- What is the impact of Aboriginal culture on effective helping support and culturally appropriate training and supervision?

## Process and method

### Background

The research design allowed for an intense focus on the experience of individual practitioners and helpers living in northern BC and the larger centres of Whitehorse, Yukon and Yellowknife, NWT and other outlying communities. Our intent in the qualitative phase was to first focus in-depth on stories through individual interviews and to gain an understanding of the wider community context in order to use participants’ knowledge and information in the development of a survey to access a broad range of helpers in the North (9). Narrative interviews were chosen for the descriptive, in-depth data collection of data. Josselson et al. (10) suggest that narrative inquiry is capable of creating a description of a historical or personal event that is rich and multilayered.

It offers a means of understanding the past in order to go beyond it, of finding the articulation between the influence of external factors and the individual's initiatives. (11)

Blustein et al. highlight the explanatory nature of this type of inquiry and its capacity to deepen and understand a participant's lived experience, noting that narratives are “particularly informative to the psychology of work for individuals who have been outside of the mainstream of career development discourse” (12). Career narratives, in particular, have the ability to identify aspects of the social realm that have enabled or constrained individuals (13). The preliminary analysis lists a multitude of social and geographical aspects related to northern mental health support work.

### Participants

In the qualitative phase, potential participants in BC, Yukon and NWT, were contacted through letters and e-mails to agencies, First Nations governments, non-profit societies and private practitioners who advertised publicly. Snowball sampling emerged after the initial sending out of information, a phenomenon that evolves in small communities where word-of-mouth communication is common. The research team travelled to the selected territories and province over two summers and one winter to conduct the initial interviews and then do follow-up contact. Interested participants were contacted by the principal investigator to set up the interviews.

Twenty northern practitioners, including both formal and informal mental health and wellness workers, took part in the in-depth interviews. Due to the need to protect participants’ anonymity during the qualitative phase in contexts where just a few details could potentially identify helpers in small communities, very general demographic information is provided. Participants ranged in age from mid-20s to 65, representing 15 women and 5 men. The formal mental health and wellness practitioners included counsellors and psychologists. The participants who were included in the informal category included professionals in other fields who were providing mental health support such as nurses and social workers, although such work was not part of their official job description, and paraprofessionals who work in child and youth care, corrections and family support positions.

Participants were interviewed by the principal investigator in a place of their choosing, ranging from offices, favourite community spots and cafes. Graduate students who were working as research assistants were included in the majority of the interviews as a part of the mentoring process and added their lenses for input on the immediate initial coding. Their presence in the interviews allowed them to bring to life each interview during the transcription and analysis process.

### Qualitative data analysis

The research team, consisting of 3 faculty researchers, 1 research associate and 3 graduate research assistants took part in Consensual Qualitative Research (CQR) analysis. CQR is a rigorous method that allows research teams to examine data, to bring a variety of opinions to each decision and to come to consensus about the meaning of the data so that the best possible construction is developed for all of the data (14). Data analysis by the research team generally involved 3 central steps: (a) clustering data into topics in order to segment interview data into domains; (b) summarizing core ideas that capture each participant's perspective and meaning; and (c) constructing common themes from each participant story and then across participants (14).

The results of the 7 analyses of the data were shared at a research gathering that resulted in critical reflection about common and divergent themes. The principal researcher and research associate then worked through all 7 analyses, defining common categories and themes, and using selections from each researcher in order to ensure that everyone's analysis was represented in the final consensual summary. The researchers later verified that they believed their analysis was represented in the first summary of categories and themes. As another verification check in the analysis, we sent participants their transcriptions and a list of individual quotes to receive permission to use. The final step is to send participants the summary for feedback and final verification and then work through a meta-analysis.

## Preliminary results

### Categories

Participants expressed specific experiences directly related to their role in mental health support based on their context of working in northern BC, Yukon or NWT. Seven broad categories are currently identified pending final feedback from the participants on the research summary, comprehensive categories that hold the main ideas expressed in the participant interviews (Table I).

**Table I:** Preliminary categories, informal and formal mental health support

Research question	Categories
What are the life-career issues, supports, challenges, and barriers for formal and informal helping practitioners in northern communities? What is the impact of Aboriginal culture on effective helping support and culturally appropriate training and supervision? What supports and resources would enhance the effectiveness and longevity of such workers?	Northern practice Community Insiders/outsiders Northern challenges Ethical issues Cultural context Sustaining northern practice

### Northern practice

Collaboration was defined as a key component to successful northern practice. Cohesive work environments were characterized by open communication, trust and support among colleagues. A team approach in agencies and across disciplines was described as enhancing the health and wellness of practitioners and promoting best practice standards including the continuity of client care. The lack of collaboration with formal mental health services was a concern for some of the practitioners. Confidentiality was viewed as creating barriers in working collaboratively across communities and providing continuity of care because client care plans are not shared with support systems in the clients’ home communities. The little to no sharing of information was described as a double-edged sword because it is meant to protect clients but was also viewed as impeding the continuity of care.

Northern practice was consistently defined as broad, generalist and eclectic because of the diverse population and range of client issues. Participants found it challenging and often overwhelming to work with individuals presenting with multiple diagnoses. Practitioners struggled with knowing where to start with clients who have long histories of abuse by multiple offenders. For example:

When you train, you normally talk about a client who comes in with depression you know a, b, c and you work through what would happen. But when your client walks in and you're like I can't distinguish one issue from another, it's like the comorbidity, but it's everyone you know and complex trauma (10111101).

Participants confirmed that accessing mental health support is often viewed negatively and there has not been enough of a shift away from the hidden nature of mental health issues. One participant questioned how the mindset towards counselling can be shifted to be a source of pride rather than something shameful.

For informal helpers, northern practice included the politics of professionalism. The message front-line workers reported receiving is they are not counsellors and they do not have the skills to work with complex issues. One participant addressed this issue:

Well you do as much as you can, but like we're not supposed to open a can of worms, right. So if something is really bugging the kids and you ask them once you have to leave it alone because if you open it, then you're counselling (10081301).

### Community

With smaller populations in communities, the requirement to connect with community was considered part of northern practice. The participants emphasized the time and consistency necessary for helpers to build trust in a community:

Being in the small community and being able to build relationships with other agencies, I think that's something that is more difficult in other places, so I think that's great …. They know who you are, they know where you are, they know what you do because we're small enough (10071601).

Mentorship was identified as a key component for practitioners working in isolated communities. The participants recommended getting acquainted with colleagues and working closely with community advisors and Elders to best address the community needs. Practitioners interviewed strove to represent the community and help people get the supplies and resources they needed.

The connection with community was described as meaningful and life changing for some of the participants. Practitioners discussed their relationships with other community members and the deep emotional connection to what was happening within their community.

You have to find the medium that works for you and maybe that community worked for me and I worked for that community … we could create things because of context, place, fate, time you know that kind of piece (10072202).

### Insiders/outsiders

In concrete terms, an “insider” represents a helper who is considered a long-standing member of the community while an “outsider” is a helper who comes into a community from some other place. In many of the interviews, the constructs of the insider and the outsider emerged as distinct from one another and were discussed in terms of the pros and cons that are associated with helpers who are in either position.

Outsiders were described as often lacking an understanding of the broader contexts in which their clients’ issues are positioned. The participants explained that outsiders who come into communities in helping roles bring with them the advantage of a fresh perspective. One participant captured this phenomenon as he described his own experience of being an outsider:

I think that's part of my strength because I am an outsider in a lot of ways, then I can see things with a different perspective. I think that would definitely be an asset to any northern organization to have an outside perspective because it's very easy just to lose sight of the bigger picture (10110801).

Insiders are often well respected in communities and have established trust over years of being consistent and visible within the community. However, the burden of practice was seen to be very heavy for insider helpers according to the participants because of the degree of enmeshment and expectation that is placed on these helpers. Their connections within their community, through both historical and current associations, was often so wide spread that any client an insider worked with in a helping context, were also connecting that helper through a number of other channels.

### Northern challenges

Many of the challenges of isolated practice have been identified in previous research (15–17). Aspects of social, professional and personal isolation directly linked to geographical components were again discussed by the participants. One of the biggest challenges in the North identified by the practitioners is that the mental health issues are endemic and so deep into the core of the communities. They described a lack of understanding of mental health issues resulting in stigma and isolation.

The high turnover rate in the helping professions was described as an on-going challenge in the territories and northern BC. One practitioner provided an example to illustrate this issue:

But constantly having to adapt and to adjust and change and that can be stressful you don't even realize it after a while. Now we have someone who signed on for a year. A year … after a year she'll be gone, good bye. Before her there was another psychiatrist, signed on for a year. And you know someone was saying to me change is good. I said not always (10111403).

Practitioners were often unable to meet the needs of the clients because of large caseloads and administrative demands. Both informal and formal practitioners addressed concerns regarding the lack of clinical supervision in the North.

### Ethical issues

The challenging concept of confidentiality became a theme of its own, as did the discussion around codes of ethics and what has to be changed to make those codes appropriate to northern mental health and wellness support. The codes were described by some participants as being from an old model that does not work in the North, with the need for revision for northern practice. It was suggested that the current codes provide a structure for ethical practice that is more useful for young or new practitioners.

In small, isolated communities confidentiality is a major concern, with practitioners unable to express their cases with anyone except those directly involved. This situation was described as fear-based because some people could be trusted and others could not. One participant explained:

Holy smokes, I understand the confidentiality piece, absolutely but the system really stinks sometimes because it's not that I'm breaching confidentiality. I don't want to breach; I want to work as a team …. (10111401-2)

It is common for northern practitioners working in isolation to have no one to consult with so the participants stressed the importance for all helpers to be trained in ethical standards of practice.

### Cultural context

The participants in this study identified a number of elements that are central to culturally appropriate practice in the North. The term “cultural sensitivity” was used to define an approach which incorporates awareness and acceptance of cultural differences as well as openness to learning about Aboriginal cultures and each client's cultural context. Awareness of an Aboriginal orientation suggested by participants is having some knowledge about the beliefs and values of Aboriginal cultures and understanding the diversity of these beliefs and values.

Another vital component shared by participants was the need to understand the transmission, depth and prevalence of the historical trauma that plagues Aboriginal peoples. The idea that the trauma is prevalent and pervasive was discussed throughout the interviews. As well, participants suggested that understanding context involved recognizing that all client issues are connected in some way to the loss of cultural identity and language.

### Sustaining northern practice

The theme of connection and relationships highlights sustaining factors of reciprocity, connection and community. The participants repeatedly discussed meaningful relationships with colleagues, clients and community members. Some of the participants described feeling very supported by their colleagues, with peers serving as practitioners’ main supporters.

The passion and commitment for the work was driven by the connections with clients and community. One practitioner described it as, “the love you have for a feeling, a place, people” (10072202). The practitioners were passionate about advocacy at all levels including advocating for clients and community, for their profession, and for changes in systems. One participant explained:

The advocacy piece is huge because when you're so concerned with the level of racism, discrimination, oppression, poverty then you have to do something. And that's where it went to you know that's where it went to (10072202).

Resilience was also identified by long-term practitioners who have continued to do the work for many years while colleagues have quit, changed professions, or gone on education or sick leave. For these practitioners, living in the North was described as providing opportunities to meet interesting people and work with diverse cultures.

## Conclusion

The skills and wisdom held by the participating practitioners is evidence of the requirements for doing this extreme form of mental health support in some of the most challenging conditions in North America. Mental health and wellness support in isolated communities with high professional visibility and limited support demand-specific qualities from the people who provide such support. Research and community development focused on mental health may help to alleviate the workload of such practitioners.

## Future research

In the beginning of the qualitative interviews, we had to redefine how we used the term “mental health” as informal helpers appeared to see the term in a more narrow Axis II interpretation, so we added the term “wellness”. In future research, we will define mental health in the broadest terms with participants. We wonder if the stigma of mental health issues may be tied to this narrow definition of the term in communities and whether workshops on broad mental health issues and the effects of complex trauma would be helpful.

Future research into Aboriginal views and strategies on improving mental health within northern communities would be extremely beneficial. More in-depth explorations of the situation of a mental health insider and informal supporters would also potentially improve services. In our research we will work to be more specific in defining who informal practitioners might be and what type of support they provide.

## Limitations

In conjunction with redefining the term “mental health”, we realize that we were not as successful in accessing those practitioners who provide informal mental health support, particularly Aboriginal informal mental health supporters. This type of recruitment would involve the use of community connections and the balancing of dual relationships. We also realize that many informal practitioners are doing essential mental health support but may not define their work in this way, related to a more narrow definition of mental health.

## Research reflection

Northern mental health and wellness practice could be viewed as living in a land of uncertainty where individuals, ethical practice and communities were evolving as issues of practice and culture were worked out in northern settings. The external components of geography, community, resources and funding all appear to contribute to the internal struggles of practitioners. Many of the practitioners who participated in this study have found ways to overcome the biggest challenges of northern practice, yet the limitations of small populations and lack of resources in small communities to adequately address mental health support were identified as existing. With issues contributing to lack of mental health and wellness defined by participants as endemic and deep into the core of many communities, communication between practitioners may be a positive step in defining the commonalities and the differences found in various northern settings and in sharing strategies and local knowledge that may have relevance to other communities and practitioners. Empowering communities by building community capacity to educate, supervise and support formal and informal mental health workers is a long-term goal of many of the participants and the authors.
